# “*It’s like a car that doesn’t like gasoline*” - a qualitative study of siblings’ understanding of anorexia nervosa in childhood: perspectives from siblings and parents

**DOI:** 10.1186/s40337-025-01263-5

**Published:** 2025-10-21

**Authors:** Amalie Schumann, Torun M. Vatne, Louise Dalton, Elizabeth Rapa, Krister W. Fjermestad

**Affiliations:** 1 Department of Psychiatry, University of Oxford, Oxford, United Kingdom; 2 Department of Psychology, University of Oslo, Oslo, Norway; 3 Frambu Resource Centre for Rare Disorders, Siggerud, Norway

**Keywords:** Siblings, Parents, Anorexia nervosa, Family perspectives, Communication, Qualitative research

## Abstract

**Background:**

When adolescents develop anorexia nervosa (AN), this impacts the family system and puts healthy siblings at risk of mental health problems. Siblings need support and age-appropriate information about the diagnosis to prevent negative mental health outcomes. However, evidence-based support for siblings is limited. The current study aimed to explore siblings’ perceptions of AN and parents’ beliefs about siblings’ understanding expressed within an intervention programme for siblings and parents.

**Methods:**

This qualitative study employed a hybrid approach, integrating deductive thematic analysis using the common sense model of self-regulation as a coding framework with inductive thematic analysis. The data materials comprised (1) interviews conducted by clinicians with siblings about the AN diagnosis, (2) siblings’ understanding of AN as expressed in sibling groups, (3) parents’ beliefs about what siblings understand expressed in parent groups, and (4) parent-sibling conversations about AN. Video and audio recordings of the data were transcribed and analysed. The sample comprised nine siblings of European descent, aged 8 to 15 years, and their parents. All siblings had a sister with clinically confirmed AN.

**Results:**

The siblings had limited knowledge and expressed uncertainties about AN across the five themes identity (label and symptoms), causes, consequences, treatment, and timeline. In the inductive analysis, two additional themes were identified. The first, *Parental perspectives on siblings’ understanding*, had two sub-themes: *AN as a confusing and complex disorder*, and *Discrepancy between siblings’ understanding of AN and parents’ beliefs about their understanding*. The second theme was *Barriers to communication about the diagnosis*.

**Conclusions:**

The results extend knowledge about informational support needs in siblings of adolescents with AN. Insights into what siblings and parents of adolescents with AN share about the diagnosis in different contexts can be used to guide the adaptation of interventions and policies.

**Trial registration:**

ClinicalTrials.gov NCT04056884.

## Abstract (plain language summary)

When a teenager develops anorexia nervosa (AN), it affects the whole family, including siblings. Siblings need support and age-appropriate information about AN to prevent negative effects on their mental health. However, there is little research on how to best support siblings. This study examined how siblings understand AN. Interviews with siblings and conversations in sibling and parent groups as well as dialogues between siblings and parents, were analysed. The study involved nine siblings aged 8–15 years, and all had a sister with AN. The results showed that siblings had limited understanding of AN, including its name, symptoms, causes, treatment, and prognosis. Parents often believed that AN was difficult for siblings to comprehend, especially its uncertain prognosis. There was a gap between what siblings knew and what parents believed they knew in some of the families. The interviewees reflected on reasons why it was difficult to talk about the illness within the family. The study highlights the need for better informational support for siblings.

## Introduction

Anorexia nervosa (AN) is a complex mental health disorder associated with significant physical and psychological problems, with one of the highest mortality rates among psychiatric conditions [1, 2]. AN also impacts the family system, including siblings [3–5]. Two systematic reviews have investigated the experiences of siblings of individuals with eating disorders (EDs) [6, 7]. The most recent review retrieved and synthesised 21 studies, reporting increased psychosocial risks among siblings, including higher levels of depression, reduced quality of life, difficult feelings about food and body image, and lower academic functioning [7]. There is a gap in the literature regarding risk and resilience factors that may affect siblings’ mental health and well-being. Recognised potential factors include illness duration, access to support, family functioning, and perceived negative interpersonal interactions resulting from having a brother or sister with AN [5, 8, 9].

The impact of AN on parents may contribute to the distress experienced by siblings. Siblings of children with AN have reported that the family is their primary source of emotional support and have also reported that it is difficult to access support outside the family [10]. At the same time, parents have expressed feelings of neglecting their other children due to the demands of caring for the child with AN [11]. Compared to families in community samples and families where a child has diabetes, mothers of children with AN have reported significantly greater family conflict, reduced parental couple alliance, and higher depression symptoms [3]. This is important for siblings’ health, since mental health challenges among parents are a known risk factor for negative outcomes in children, such as impaired physical and mental health [12, 13], while reduced parental alliance and heightened family conflict can cause parents to be less responsive to children’s needs [14].

Few evidence-based interventions are available for siblings of children and adolescents with AN, and siblings are rarely involved in AN treatment [15–17]. Family-Based Treatment (FBT) is the most effective documented treatment for AN in adolescents [18, 19]. The theoretical foundation of FBT is that since the adolescent is embedded within the family system, recovery requires the involvement of family members, especially parental involvement in the weight restorative phase [20]. In the FBT manual, siblings are described to play an important role in family therapy sessions, during meals, and in providing support throughout the treatment [20]. However, siblings have reported feeling excluded and have expressed a desire for greater support in FBT sessions, and they are rarely actively involved in practice [16, 21, 22]. Importantly, even when siblings are included, the available evidence suggests that FBT is not associated with benefits for siblings’ mental health. Van Langenberg, Sawyer [8] reported that siblings of patients with AN undergoing FBT reported significantly higher emotional difficulties compared to population norms, that did not decline from pre- to post-treatment. Parents did not report significantly heightened emotional difficulties among siblings at pre-treatment, only at post-treatment, suggesting that parents may have become more aware of the impact of AN on siblings during the course of FBT [8].

Inpatient treatment introduces additional challenges, as parents may struggle with decisions about siblings’ involvement. In family-based inpatient treatment where family members are admitted alongside the patient with AN, both parents and patients with AN have reported that siblings gain limited benefits from this treatment approach [23]. Siblings have reported feeling supported during inpatient family treatment, but they faced ongoing difficulties after discharge, as no further support was offered to address their needs [24]. Hence, although FBT is effective for AN, it does not seem to benefit siblings in its current form.

Overall, research suggests that siblings require more tailored support than what FBT provides in inpatient and outpatient settings. Siblings have reported that they are rarely offered professional support and often avoid seeking help due to barriers such as stigma, a lack of information, and doubts about the severity of their needs [15]. The initial phase of the illness has been reported as being especially difficult [15], and there is a strong desire among siblings for greater support, including individual counselling, school-based education, parental guidance, and group interventions [21, 25, 26].

Qualitative studies consistently report siblings’ need for information about the illness. Siblings have described AN as difficult to understand and reported receiving little information about the diagnosis from either their parents or healthcare professionals [21, 22, 26]. Within the ED literature there is limited understanding of the specific information siblings require and how unmet informational needs affect them. In a qualitative study, siblings reported that a poor understanding of AN led to feelings of fear and anger, and that increased understanding helped them cope better (e.g., by externalising the disorder), and maintain a better sibling relationship [10]. Insights from research on other conditions, such as psychotic disorders and chronic health issues, provide further context. Findings from these fields suggest that insufficient knowledge can lead to uncertainty and adjustment difficulties among siblings, while increased understanding through psychoeducation supports the development of effective coping strategies [27–29]. Hence, addressing siblings’ informational needs is important.

The common-sense model (CSM) provides a framework for understanding how people perceive and manage health issues [30]. A key component of the model is the cognitive representation of illness, which influences how individuals cope with illness [30, 31]. The cognitive illness representation comprises five dimensions: (1) Identity: The illness label or name and its symptoms, including changes in function and visible signs of the illness. (2) Timeline: The perceived duration of the illness, both with and without treatment. (3) Cause: The single or multiple factors believed to cause the illness, such as injury, infection, or genetic factors. (4) Consequences: The expected and perceived physical, personal, social, and economic effects of the illness. (5) Cure/control: Beliefs about whether the illness can be cured or managed either by the body’s defence system and/or treatment [31].

The CSM was designed to capture perceptions of physical illnesses, but there is growing interest in applying the model to explore perceptions of mental disorders, including AN [32, 33]. However, limited research has focused on children’s perceptions of mental illness, and no studies have been identified that explore siblings’ cognitive perceptions of AN.

The CSM has been applied in other sibling populations and in studies exploring parents’ perceptions of AN. In a study examining siblings’ understanding of rare disorders (i.e., disorders that occur in fewer than 1 in 2000 individuals), researchers found that children primarily discussed the symptoms and causes related to the illness dimensions and exhibited a high frequency of misconceptions about these two dimensions [27]. A lack of available information about the disorder contributed to uncertainty among the siblings [27]. Recently, a systematic review of qualitative studies applied the CSM to investigate parents’ perceptions of their child’s AN diagnosis [34]. Parents viewed AN as a serious health threat, describing it as uncontrollable, difficult to understand, long-lasting, and associated with negative outcomes. Many also felt personally responsible for their child’s condition. These illness perceptions were linked to feelings of fear, anxiety, shame, guilt, loneliness, and depression [34]. The findings suggest that the CSM can be useful for understanding factors contributing to parents’ distress and the challenges of caring for a child with AN. Exploring siblings’ cognitive perceptions of illness could build on these findings and provide a more comprehensive understanding of how the family as a whole perceives and copes with AN, potentially informing family-based interventions.

The current study aimed to explore siblings’ knowledge and perceptions of AN expressed across different contexts within an intervention programme: during interviews with clinicians, within sibling groups, and in parent-sibling dialogues. Parents’ perceptions of siblings’ understanding of the diagnosis were explored through an analysis of their contributions in parent groups. By mapping out potential informational needs among siblings, the study aims to inform the field on how to tailor interventions for siblings.

## Method

### Design and setting

The current study is part of an ongoing randomised controlled trial (RCT) investigating the effectiveness of the group intervention SIBS [see Fjermestad, Silverman [35] or the full study protocol], conducted in specialist and primary health care services across eight sites in Norway. SIBS is a manual-based intervention targeting siblings and parents of children with various chronic disorders. The intervention comprises five sessions with separate group sessions for siblings and parents, followed by joint parent-sibling sessions. The primary goals are to improve parent-sibling communication and equip parents to provide better emotional and informational support to siblings [36]. The data in the current study are drawn from interviews pre-intervention, and two sessions in the intervention where the thematic topic is “the diagnosis.” The sessions are described below. The interviews and group sessions analysed in the current study took place and were recorded between October 2021 and June 2022.

The data was analysed qualitatively using a hybrid approach of deductive theory-driven and inductive thematic analysis [37, 38]. The dimensions of the CSM framework was applied as template of themes, while the inductive, data-driven approach generated new themes based on the data [37, 39]. The CSM has been widely applied as a framework in qualitative research [40] including synthesis of qualitative studies of parents’ understanding of AN, and exploration of illness perception in other sibling populations (siblings of children with rare disorders) [27, 34]. The hybrid approach of theory-driven deductive analysis and data-driven inductive analysis was chosen as it allowed for the structured application of the CSM while remaining open to emerging insights from the data that were relevant to the research question but did not fit within the framework [41, 42]. The current study is reported according to The Standards for Reporting Qualitative Research (SRQR) [43].

### Characteristics of participants and sampling procedure

The participants were siblings and parents of adolescents diagnosed with AN who were purposively sampled from the complete data set of the RCT. The inclusion criteria for the RCT study from where the data is drawn are: (1) being the sibling of a child aged 0 to 18 years diagnosed with a chronic disorder who receives specialist and/or primary care health services. The chronic disorders included are diabetes, cancer, congenital heart disease, eating disorders, neurodevelopmental disorders, and rare disorders. (2) Sibling age from 8 to 16 years. (3) One parent able to attend the intervention. The exclusion criteria for siblings are: (1) Being enrolled as patients in specialist mental health services. (2) Being diagnosed with any of the inclusion disorders. The participants were recruited through multiple pathways, including sites’ clinical databases, adverts and posters in waiting rooms, patient user forums, and social media.

### Data sources

#### Sibling knowledge interview (SKI)

The SKI is a structured interview developed to assess siblings’ understanding of their brother’s or sister’s illness [44], and has been used in previous studies of sibling-focused interventions involving siblings of children with chronic conditions [44, 45]. The interview covers the name or label of the illness, explanations of its core symptoms, consequences, causes of the illness, and treatment. The interviews included in this study were conducted pre-intervention by trained clinicians and lasted between 2–7 min (*M* duration = 5 min).

#### Sibling groups

The video material used in this study covered two sibling group sessions from the SIBS intervention: In the first session, the siblings get to know each other and establish group rules (approximately 20 min). In the second session, the siblings discuss and share their thoughts about the diagnosis (approximately 60 min). During the session, the siblings formulate questions for their parents about the diagnosis, which they later discuss in parent-sibling dialogues, as detailed below. Each sibling group comprised three to six participants, whose brothers or sisters had various disorders as outlined in the inclusion criteria of the RCT study. The sessions were led by two trained group leaders. The sibling group sessions analysed in this study lasted between 64 and 95 min (*M* duration = 81 min).

#### Parent groups

The video material used in this study covered two parent group sessions from the intervention. In the first session, parents are provided with information about the intervention (approximately 20 min). The second session focuses on teaching parents techniques for listening to, exploring, and validating the siblings’ thoughts and emotions related to the diagnosis (approximately 60 min). Each parent group comprised three to six participants and was led by two trained group leaders. The parent group sessions analysed in this study lasted between 79 and 96 min (*M* duration = 88 min).

#### Parent-sibling dialogues

In the group sessions described above, both parents and siblings prepare for a joint session involving parent-sibling dialogue. In the dialogue, the sibling asks the parent their prepared question(s) about the diagnosis; the parent practises actively listening to the sibling, exploring the sibling’s thoughts and emotions related to the question, and validating the sibling’s thoughts and emotions. Each sibling-parent pair sits separately from the other participants, and a group leader provides brief input during the conversation focusing on the parent’s communication techniques (i.e., no input regarding AN). The dialogues analysed in the current study lasted between 21 and 44 min (*M* duration = 29 min).

### Qualitative analysis

The first author transcribed the audio files of the clinician-sibling interviews (SKI) and the parent-sibling dialogues. The interviews (SKI) were fully transcribed. In the dialogues, only expressions related to the AN diagnosis were transcribed excluding segments where group leaders gave feedback to the parent on communication techniques, and when the sibling and parent discussed practicalities (e.g., the duration of the conversation or travel arrangements). The videos of sibling and parent groups were watched and transcribed by the first author, with segments omitted where the diagnosis was not the topic (e.g., introductions in the groups, when group leaders provided information about the SIBS intervention, and when parents where taught communication techniques in the parent group). Identifiable information was removed or changed in the transcribed material. The material was transcribed verbatim.

The data was coded top-down applying the five a-priori themes in the CSM framework of illness perception: Identity, Cause, Timeline, Cure, and Consequences [30, 31, 38]. Data that did not fit within the framework but was interpreted as relevant to the research question was coded into new themes following the steps of inductive thematic analysis: (1) familiarise with the data, (2) generate initial codes, and (3–5) search for, review, and name themes [37, 39]. The data analysis was a reflexive process involving iteration between phases.

All authors read the transcribed material to get a grasp of the data. AS coded the data applying the CSM framework. Material that was considered possibly relevant to the research question, but considered not applicable to the CSM framework, was coded by AS into new themes. The content (quotes) of initial codes and later derived themes, both the pre-defined and the data-generated themes, were carefully reviewed by all authors throughout the data analysis process. Meetings between the authors were conducted to discuss and revise the themes. The data was transcribed in MS Word and coded using the NVivo-14 software. Research journaling was conducted by AS within NVivo, noting down reflections, ideas and impressions of the data during the analysis process.

### Ethics

The Regional Committee for Medical and Health Research Ethics South East approved the larger RCT (#2018/2462). The approved study protocol described that qualitative analyses of video and audio recordings were planned as part of the study. Parents provided parental consent for children’s (< 16 years) participation, and children aged 12 years and older gave assent to participate. Adult participants (the parent participants) gave informed consent for their participation. The interviews and parent-sibling dialogues were audio-recorded, and parent and sibling groups were video-recorded. In all instances, a recording app was used, and the files were automatically transferred to a secure database approved by the relevant data protection agency.

### Trustworthiness and reflexivity

Trustworthiness in qualitative research, as outlined by Lincoln and Guba [46], is established through credibility, dependability, confirmability, and transferability. Credibility: The study employed well-established qualitative methods, including deductive thematic analysis using the CSM framework [38, 40] and inductive thematic analysis [37, 39]. The combination of inductive and deductive analysis is described and used in qualitative research across academic fields [41, 42]. Dependability: Data were collected from multiple sources at different time points and in various formats, including clinician-sibling interviews, sibling group discussions, and parent-sibling dialogues. Parents’ perspectives in group discussions further contextualised siblings’ understanding of AN. The different sources of data enabled data triangulation, checking if the same perspectives were evident across contexts [47]. Confirmability: All researchers reviewed the transcribed material, and themes were iteratively refined through discussions in data analysis meetings. AS attended all meetings, alternating between the British (LD, ER) and Norwegian (TV, KF) research teams to ensure diverse perspectives informed the analysis. This enabled triangulation of perspectives of the researchers [47]. Transferability: The potential influence of context on the qualitative findings is acknowledged. Relevant contextual details that were collected about the participants, setting, and research process are provided. However, contextual factors such as participation in an intervention may have shaped participants’ responses. This is further discussed in the limitations section.

Reflexivity: The authors aimed to remain mindful of how analysis of the material could be influenced by preconceived beliefs. Participants shared sensitive information about themselves and their families, hence, it is important that the results reflect their experiences and are recognisable to them [48]. However, in line with the constructivist paradigm, it is acknowledged that beliefs, values, and judgments inevitably shape how themes are created and interpreted [49]. The authors represent two research teams from Norway and the United Kingdom. All authors are engaged in research on child and adolescent mental health. Four authors are clinical psychologists with varying levels of clinical experience and training in different countries. Two researchers (KF and TV) developed the intervention from which the data were drawn, and KF was a group leader in one of the groups.

The authors engage in research on family communication about illness and sibling interventions, with a shared belief that children benefit from age-appropriate information to promote coping and prevent negative mental health outcomes. While this view is grounded in existing research [50], the authors recognise that it may shape interpretations of the data and findings. To maintain a reflexive approach, the analysis was conducted collaboratively. The authors held regular meetings to examine interpretations and consider alternative perspectives, increasing the likelihood that the findings were grounded in the data rather than shaped by pre-existing assumptions.

## Results

### Characteristics of participants

The sample comprised nine siblings (6 (67%) girls; 3 (33%) boys) who participated in six different sibling groups. Their mean age was 10.9 years (*SD* = 2.8; *range* = 8–15). The siblings participated with one parent, two with their father and seven with their mother. The nine siblings represented five unique families. Three families had several children participating in the intervention. The siblings had a sister who had been assessed and received treatment for a clinically confirmed AN diagnosis in Child and Adolescent Mental Health Services. The mean age of the sisters with AN was 14.2 years (*SD* = 1.1; *range* = 13–15). The families were characterised as follows: The average sibship size was 3.6 children (*SD* = 1.9; *range* = 2–7). Two were older siblings of the child with AN, and seven were younger. Parents’ mean age was 47.2 years (*SD* = 4.2; *range* = 41–53). Regarding socio-economic status, all parents reported to have completed higher education after high school. One parent (10%) reported up to 4 years, and nine (90%) reported ≥ 4 years post high school education. All families reported European descent.

### Qualitative results

First, the results of the theory-driven thematic analysis using the CSM framework to examine siblings’ understanding of AN are presented, including the five themes: identity, cure, cause, timeline, and consequences. Next, the results of the inductive thematic analysis of additional emerging themes are presented, including perspectives from both parents and siblings. These results include the themes “Parental perspectives on siblings’ understanding” divided into two sub-themes: (a) AN as a confusing and complex disorder, and (b) Discrepancy between siblings’ understanding of AN and parents’ beliefs about their understanding, and “Barriers to communication about the diagnosis”. All themes are supported with illustrative quotes, along with pseudonyms (fictional names) and ages of the siblings.

### Siblings’ understanding of AN

#### Identity

Siblings’ knowledge about their sister’s AN diagnosis varied, both in terms of labelling and explaining symptoms. Three of the nine siblings did not accurately name or label the disorder during the interviews. For example, one sibling was asked what their parents call the diagnosis after stating she did not know the name: “*They don't call it anything, I don't know what they call it, I… They just say that she has a diagnosis*” (Emma, 9 years). Some siblings’ understanding appeared to come from signs they observed or overheard rather than direct information from parents or healthcare providers. One sibling guessed the disorder might be called “Ed” [i.e., the male name] because her sister wore a bracelet that said “*fuck ED*” (Nora, 9 years).

When asked to describe the signs of AN and affected body parts, some siblings demonstrated limited understanding, misconceptions, and gave vague answers: “*I think it’s something about eating, that she can’t eat properly or something”* (Nora, 9 years). Siblings also gave answers indicating that their knowledge did not come from parents or healthcare providers: “*Ehm… I know that her hair got very thin and uneven… And we’ve learned a bit about it in school. My teacher said that it could cause your period to stop, but I don’t know if that happened to her”* (Lily, 14 years). Misconceptions were also evident among some siblings: *“[The affected body part is] the mouth”* (Leo, 9 years).

Other siblings demonstrated broader understanding of AN. These siblings described the disorder as involving a problematic relationship with food, with challenges around perception of weight and finding food complicated. They recognised that the disorder is related to the brain and affects the whole body: “*It seems like it’s the entire body [that is affected], and they might become tired […]. And maybe a bit in the brain because the eating disorder might be based there*” (Ida, 9 years). Another sibling compared his sister to “*a car that doesn't like gasoline”* (Adam, 14 years) to illustrate the impact of AN on health and functioning.

In both the sibling groups and the parent-sibling dialogues, knowledge about the name of the illness and symptoms were not elaborated on, but some siblings mentioned observable emotional reactions and behaviour as signs of the diagnosis: *“[she] cries a lot and screams a lot.”* (Leo, 9 years).

In the sibling-parent dialogues, few siblings prepared questions related to the identity dimension, for example asking the parent where the diagnosis is located in the body, whether it is all over, or *“in the thoughts in a way”* (Leo, 9 years). However, several siblings discussed how the disorder manifests during conversations with their parents: *“It’s not fun that she’s a bit angry all the time. It’s like it’s impossible for her to become happy with that eating disorder […] and she scolds us a lot”* (Lucy, 8 years).

#### Cure

The siblings expressed uncertainty about the treatment process in the interviews: “*I don’t know, doctors… she’s at a place. I’m not entirely sure”* (Emma, 9 years). Others suggested that avoiding the topic might be helpful for recovery. For instance, when asked what others can do to help, one sibling said: *“Actually, just acting normal and not talking about it”* (Leo, 9 years). Another sibling explained that he had been explicitly told not to talk about the diagnosis but had mixed feelings about this: *“I've tried to keep distance from it. I've been asked by mum and dad not to talk too much with her about it since she’s a bit touchy… But I don't want it to be taboo, in a way, because then it becomes more difficult”* (Adam, 14 years). Distractions or avoidance were also mentioned in relation to mealtimes: “*Doctors, or all people who meet the person [with AN], can do or watch something fun together when eating, so that you can think about something else than eating”* (Mia, 12 years).

A few siblings showed a well-informed perspective, recognising that changes in food intake and involvement of parents are central to treatment. They mentioned concepts like meal plans, “*food as medicine*,” help from parents to track calories, and the importance of eating more and different types of food. Some siblings discussed the need for changes in cognition and self-concept as part of recovery. They talked about addressing a “*disrupted mindset*,” and learning to love oneself again. They noted that therapy involves helping their sister feel safe and encouraging her to choose to eat on her own. However, even among those with more knowledge, there was still a sense of uncertainty about the details of the treatment. For example, when asked what teachers, doctors, and parents can do, one sibling stated: “*maybe [others can] convince her to eat*?” (Lucy, 8 years).

In the sibling group, uncertainty about the specifics of therapy was evident. For example, when one sibling was asked about the treatment of AN and what she knew about her sister’s treatment sessions, she replied: *“I know that she’s here [Child and Adolescent Mental Health Services] once a week or something. I think that’s the treatment she receives now […] I think she talks to a person about, not about her eating disorder, but everything else that’s going on in her life”* (Mia, 12 years).

In the parent-sibling dialogues, some siblings had questions about cure. They wondered how you can be certain that someone has recovered from the disorder and how long their sister would need to maintain a sufficient food intake before being considered recovered.

#### Causes

The siblings displayed varying levels of understanding about the causes of AN in the interviews. Some siblings expressed simple attributions, such as *“you get it by stopping eating”* (Lucy, 8 years). In contrast, other siblings communicated a combination of factors that may contribute to the development of AN. These included cultural and societal pressures, social influences like peer pressure and bullying, and developmental aspects such as age and the challenges of adolescence: *“It can be many things these days. Social media and stuff like that. And peer pressure. And she’s a teenager, so it may become extra difficult due to that”* (Adam, 14 years). Another sibling highlighted the many possible causes of AN for her sister: *“She was going to start in a new class, so she may have been a bit worried about how people in the new class would perceive her. And she watched TikTok and YouTube and wanted to exercise. She exercised a lot during the summer vacation […]. Locked herself in her room and exercised. And she does [aesthetic sport, anonymised]. So, when she started training, it probably just got worse*” (Lily, 14 years).

Despite some siblings’ awareness of plausible factors that could contribute to developing AN, many others experienced a sense of uncertainty and confusion about the causes of their sister’s AN. The uncertainty and confusion were more evident in the sibling group and conversations with parents compared to the interviews: “*I actually don’t know how she got it… I've talked with mum and dad about it, but I really don't know why someone develops it, or why she got it specifically. There are probably many things that have played a part, but I don't really know why… I’m actually wondering about that myself”* (Eva, 15 years).

In the parent-sibling dialogues, the siblings’ questions often focused on the causes of AN: *“Why did she become sick?”* (Lily, 14 years), *“What are the reasons for eating disorders? Are there other reasons than that you just stop eating?”* (Lucy, 8 years).

#### Timeline

Although the interview schedule did not address the timeline or prognosis of AN, the topic emerged as a concern for the siblings in the other contexts. In the sibling groups, they expressed worries and uncertainty about how their sister’s condition might develop over time: *“Yes, [I worry about the future], because you don’t know how it will turn out. She can get well. But because she has the disorder, she might not be able to do normal stuff… like having a family and being with someone. She might not be able to do that”* (Eva, 15 years). Further, siblings were troubled by the uncertainty of their sister’s future care as she grows older: *“Regarding the future, I have talked with dad about it, and he says eating disorders can last for some months or several years. I hope it will pass soon… in two years, she will be an adult, and then I don’t know if she will still be taken care of… If no one is watching over her, she might end up eating nothing”* (Adam, 14 years). Some siblings had misunderstandings about the prognosis: “*My sister is going to be well when she’s about 16 or 17. Then she’ll be finished with the disease […]. Dad said it will take four or three years, and she got it when she was 13”* (Leo, 9 years).

In the parent-sibling dialogues, siblings asked direct and emotionally charged questions about the timeline and potential outcomes of the disorder: *“* < *tearful* > *When do you think she will become well? And do you think she ever will become well? […] How long do you think it will take? Because I want her to become well, I want it to return to how it was before!”* (Lucy, 8 years). The siblings were also wondering about the possibility of death and chronicity: *“Will she have it forever? And can you die from it even when you receive help?”* (Ida, 9 years).

#### Consequences

When answering questions about consequences in the interview, the siblings gave few and short descriptions of the physical, personal, social, and economic effects of the diagnosis. When asked about the challenges and strengths people with eating disorders may have, one sibling replied: *“Eating and talking about it. It almost seems normal […] they are good at not getting very hungry and not getting grumpy when they are hungry”* (Ida, 9 years). Another sibling remarked: *“Can’t exercise and can’t eat. I don’t know [the strengths]”* (Nora, 9 years). Consequences, as outlined in the CSM, were addressed by the siblings in other sections of the interview. For example, when the siblings were asked to elaborate on the signs of the diagnosis, they described how their sister could isolate herself from the world or needed to stay home from school.

In the sibling group, the siblings described physical consequences during group discussions such as fainting from undernourishment, and changes in appearance: *“It was the worst during the summer vacation. She fainted a couple of times because she was undernourished […] you could see that she was getting skinny”* (Adam, 14 years). During parent-sibling conversations, siblings asked about how their sister was coping in everyday life, for example how she experienced school and how she would manage school when being away a lot: *“Will she receive some sort of extra support [in school]? […] She has missed out on so much teaching at this point. If she’s sick for, let’s say two more years, she will be away for the rest of secondary school”* (Lily, 14 years).

### Parental perspectives on siblings’ understanding

#### AN as a confusing and complex disorder

Several parents expressed that AN is inherently difficult for siblings to understand: *“It’s a diagnosis that I believe is almost impossible to understand. I cannot understand that it’s possible myself.”* Parents also recognised that siblings of different ages and maturity levels had varying degrees of understanding: *“Compared to her older sister… who is very mature and can interpret and understand, while this one [younger sibling] is a child who cannot really comprehend and understand the volatile behaviour.”*

Parents noted that siblings found it particularly challenging to understand the long-term and unpredictable nature of AN. Parents expressed that the concept that the disorder could persist for a long time was hard for siblings to reconcile, for example when compared to their own experiences with common somatic illnesses. When discussing how the prognosis is difficult for siblings to understand, one parent explained: *“When he’s sick himself, it may last for one week. But he sees that it [AN] lasts… I think it’s lots of stuff he doesn’t understand.”*

#### Discrepancy between siblings’ understanding of AN and parents’ beliefs about their understanding

In some families, there appeared to be a discrepancy between what siblings understood about AN (as expressed in sibling groups and interviews) and what parents believed the siblings understood (as expressed in parent groups). Parents often believed that siblings struggled to grasp the diagnosis and its causes. However, in some cases, siblings had more nuanced and informed explanations than parents assumed: *“He cannot understand why she doesn’t eat […] I don’t know if he has explained it to others. If he understands what the problem of his sister is.”* Yet, the sibling both explained the disorder thoroughly and provided explanations of its potential causes. In another family, a parent expressed that the sibling had knowledge of the diagnosis, but did not want to talk about it. This sibling did not state the correct name or describe the symptoms in the interview.

There were also discrepancies in what parents believed the siblings thought about the future and what the siblings expressed: “*Regarding the future […] I imagine that he believes it will be fine, maybe because we have shielded him in a way*” while this sibling expressed in the group: “*Regarding the future […] I hope it will pass soon. I can’t say she’s an adult, but in two years she will be an adult and then I don’t know if she still will be taken care of […] It’s not exactly life and death, but if she wasn’t being watched over then it may have been very serious. Or it is [very serious], but even more*” (Adam, 14 years).

### Barriers to communication about the diagnosis

In the sibling group and in the parent group, barriers to communication about the diagnosis was identified as a recurring theme, both within the family and with peers. Parents often felt limited in their ability to have open conversations about AN with their children due to both practicalities and beliefs about the siblings’ readiness to understand the situation. Some parents thought the siblings did not want to talk about it or were too young to grasp the complexities: *“They [siblings] don’t want to address it, because then it might just grow even bigger. By them not talking about it, it might go away by itself.”* Another parent highlighted time limitations due to managing multiple children and acute hospitalisations: “*It’s limited time to sit together one on one, and she hasn’t had the diagnosis for so long. […] And then it was acute hospitalisation, so to sit together and talk about, what are you really wondering about, hasn’t been possible.”* Further, some parents perceived the sibling as not being engaged or capable of understanding the situation or did not care: *“I think it’s lots of stuff he doesn’t understand. […] He’s rather thinking about Lego. I kind of think that he doesn’t care. He notices that things are really different, but I don’t think he… We talk with him, but I don’t think he has the necessary vocabulary for it.”*

On the other hand, siblings expressed their reluctance to burden their parents with their concerns, due to the emotional strain their parents were under: *“I don’t want to put my worries on them [parents]. I know that they are already going through a tough time”* (Eva, 15 years).

Some siblings reported that they seldom talked about the diagnosis with friends, either by personal preference or due to worries about how their friends might respond. One sibling shared his hesitation about discussing the diagnosis with his closest friends: *“I’m afraid they [friends] won’t be so understanding.”* (Adam, 14 years). Another sibling revealed that while she occasionally mentioned her sibling’s illness to close friends when she was struggling, she generally preferred not to bring it up: *“If I don’t bring it up, it won’t be brought up. And I think that’s nice”* (Eva, 15 years).

## Discussion

This study qualitatively explored siblings’ understanding of AN by applying the theory-driven themes of the CSM: identity, cause, consequences, treatment, and timeline. Two additional themes were identified through an inductive analytical approach: Parental perspectives on the siblings’ understanding (sub-themes: AN as a confusing and complex disorder and Discrepancy between siblings’ understanding of AN and parents’ beliefs about their understanding) and Barriers to communication about the disorder.

The results showed that many siblings had limited knowledge and uncertainty about AN across illness perception dimensions. These findings align with previous research indicating that siblings find AN difficult to comprehend [21, 22, 26]. Children’s understanding of illness is partly shaped by their parents [51, 52], and prior studies suggest that siblings often rely on their parents as their primary source of information about AN [21]. However, parents themselves have reported struggling to understand the diagnosis [53–55], which may explain why siblings in the current study particularly found it difficult to grasp why their sister had developed AN.

While this study did not examine parents’ understanding of the causes of AN, previous research indicates that parents also find the causes especially challenging to comprehend [53, 54]. Importantly, many siblings in the current study prepared questions for their parents about why their sister had developed AN and what causes eating disorders in general. This suggests that siblings may wish to engage in conversations with their parents about this topic, highlighting the importance of providing families with accurate and accessible information about AN.

The timeline aspect of AN was not directly addressed in the interview, but many siblings expressed concerns about the future in other contexts. Parents also highlighted future uncertainties and prognosis as key issues when discussing siblings’ understanding of AN in the parent groups, suggesting this is an important family concern. AN has an uncertain prognosis, particularly after discharge from inpatient care [56, 57]. Broader physical health literature indicates that uncertainty about illness and prognosis can be challenging for both children and parents, making coping difficult and potentially hindering communication [50]. The siblings’ search for a clear prognostic timeline may be a way of managing their uncertainty and worries. Conversely, parents’ own uncertainty about prognosis may contribute to this topic being avoided or omitted in family discussions.

Many siblings in the current study expressed uncertainty and vagueness in their descriptions of the psychological treatment for AN beyond increasing the food intake. FBT is a manual-based approach with clear phases (focusing on weight restoration and strong family involvement, returning control to the adolescent, and establishing healthy development), and emphasises the importance of involving siblings [20]. However, previous studies have shown that siblings are rarely involved in FBT in practice, and those who have been involved have expressed a desire for greater support in FBT sessions [16, 21, 22]. Psychological interventions can also be more difficult to grasp due to its abstract nature, unlike the concrete steps involved in increasing food intake and adhering to meal plans. In qualitative studies, parents have reported being positive towards FBT, though some feel the treatment focuses more heavily on weight restoration than the psychological aspects of the disorder [58]. Lack of sibling involvement in treatment, parents’ understanding and explanations of the therapy, and the abstract nature of psychological interventions may contribute to siblings’ uncertainty about the treatment process.

The findings suggest that some siblings acquire knowledge about the diagnosis through observations or school education, rather than direct explanations from parents or healthcare providers. Such indirect learning can lead to misconceptions and an incomplete understanding of the diagnosis [27]. In line with this, some siblings in this study expressed an erroneous belief that the disorder was located where the symptoms were visible (e.g., the mouth), based on their observations during mealtimes. Such misconceptions have also been seen in studies of siblings of children with rare disorders [27]. When children lack sufficient information, they often create their own explanations of the world around them [27, 59]. A lack of accurate information can lead to confusion, fear, and helplessness, which are feelings frequently reported by siblings in qualitative studies [15, 26].

Developmental changes in illness perception are important to consider when interpreting the results. The differences in siblings’ understanding could be related to their cognitive development, as seen in previous studies of children’s cognitive perceptions of mental illness and mental health [60, 61]. Younger children may struggle with the abstract concepts involved in understanding AN, leading to misconceptions or partial understandings. Due to the small sample size, it is difficult to determine whether siblings’ knowledge was consistently related to their age in the current study. Regardless, the child’s age and developmental ability are important to consider when communicating with children about illness within the family [50].

### Implications

The findings highlight the importance of direct, effective communication with siblings about AN. Parents and healthcare providers should be encouraged to offer clear and age-appropriate explanations about the disorder to all children in the family. This may help demystify the disorder and correct any misconceptions siblings might hold, which can hinder negative feelings and foster coping [10, 50].

The findings suggest that parents may benefit from information about how children at different developmental stages typically understand illness, how siblings may be affected, and how siblings may suppress their own feelings to avoid adding strain to the family dynamic [21].

Healthcare providers should recognise that AN is also difficult for parents to comprehend [34], and parents may need assistance in understanding and reconciling with the diagnosis to engage in honest and supportive conversations with their children.

Healthcare providers should acknowledge the important role siblings play in family dynamics. Clinicians can facilitate family meetings to address siblings’ questions and concerns, ensuring that all family members receive adequate information and support.

Additionally, siblings should be informed about the treatment for AN. The findings reveal uncertainties among siblings regarding treatment, with several expressing worries about the future and prognosis. Providing insight into treatment and available support for the person with AN may help alleviate siblings’ concerns.

This study highlights the need for future research to explore how best to implement clear, direct, and family-centered approaches to support siblings of children and young people with AN. Further research could examine the specific content, timing, and delivery methods of information provided to siblings to determine the most effective ways to enhance their understanding and coping strategies. Additionally, this study can inform the development and adaptation of sibling interventions by providing insights into the potential informational needs of siblings.

## Limitations

The study had a small sample size and included only siblings of sisters with AN. Therefore, it remains unclear how siblings of brothers with EDs understand the diagnosis. However, the inclusion of data from various contexts, along with parental perspectives, enriched the material. Additionally, there was a wide age range among participating siblings. Research suggests that perceptions of mental illness can change significantly between the ages of 6 and 11 [60], and younger children find it more difficult to make sense of unfamiliar experiences, have a more limited vocabulary, and are less practised in discussing past events compared with older children [62]. While the findings do not indicate a clear relationship between diagnosis knowledge and age, interpretation is limited by the small sample size, warranting further exploration in future research.

Furthermore, parents’ own illness perceptions were not explored in this study, yet their beliefs and knowledge may have influenced how siblings perceive AN. Additional information about the adolescent with AN, such as illness severity, treatment duration, treatment phase, and whether they received inpatient or outpatient care, could have enhanced the understanding of both siblings’ and parents’ perspectives.

Contextual factors may have shaped what siblings and parents shared in different settings. SKI is a brief interview that did not cover the diagnosis timeline. Interviews were conducted by unfamiliar adults and included specific rather than exploratory questions. Open-ended prompts generally elicit more detailed responses, particularly from young children (63). Further, the groups and the sibling-parent dialogues took place within an intervention programme in a clinical setting, and the siblings and parents were video- and audio recorded. Such aspects may have made participants more conscious of what they shared. Different answers or explanations may have emerged in a more natural setting.

Group dynamics may also have influenced discussions in sibling sessions, as contributions from other participants and group leaders may have shaped the conversations. For instance, different reflections might have emerged if the groups had included only families with a child with AN rather than a mix of different conditions. The intervention was brief, spanning just two days, which may not have allowed sufficient time to build the trust and rapport needed for discussing sensitive topics.

Lastly, siblings’ desire to protect their parents may have affected what they shared during sibling-parent dialogues. The study found that some siblings withheld their worries to avoid increasing their parents’ emotional burden, which may have influenced the information gathered from the dialogues.

## Conclusion

To the best of our knowledge, this is the first study to explore siblings’ illness representation of AN. Previous qualitative studies have primarily focused on siblings’ experiences and emotions related to having a brother or sister with AN, rather than their cognitive perception of the diagnosis itself. The results provide insights into siblings’ knowledge gaps and uncertainties across different contexts, parents’ beliefs about their understanding, and communication barriers both within the family and with peers. The findings contribute to a growing understanding of the broader family context in which AN exists and highlight the need for interventions that support all family members, including siblings. Parents should receive support in having age-appropriate and open conversations with their children. Healthcare providers should emphasise the importance of involving siblings in discussions about AN and providing them with appropriate resources to understand the disorder.

## Data Availability

The data supporting the findings of this study are not openly available due to reasons of privacy and confidentiality of the participants. Data are located in controlled access data storage at the University of Oslo.

## Abbreviations

AN: Anorexia nervosa
ED: Eating disorder
FBT: Family-based treatment
CSM: Common-sense model
RCT: Randomised controlled trial
SKI: Sibling knowledge interview
